# 

**Published:** 1882-08

**Authors:** 


### CONTENTS.

ORIGINAL:

Ligation of the Femoral Artery for Traumatic Aneurism. By George J. Grimes, M.D. .................. 641

Amputation for Traumatic Gangrene of the Foot. By C. A. Simpson, M.D. .................. 642

The Recent Vaccination in Atlanta. By John H. Logan, M.D. ........ 646

SOCIETIES:

The Atlanta Medical Society—Abscess in the Abdominal Walls—Acute Pneumonia followed by Pleuritis—Capillary Bronchitis—Neuralgia .................. 662–664

EXTRACTS:

Treatment of Cicatricial Contractions—Hahnemannism, otherwise called Homœopathy—Laceration of the Perineum—The Cure of Hernia by the Antiseptic Use of the Animal Ligature—Dengue in Columbus, Ga., in 1866—Local Anæsthesia in Neuralgia—Pompeian Surgery—Acute Tonsillitis—Foreign Body in the Cranium—Urethral Caruncle and Cystitis—Augusta Health Board—The Thermometric Bureau—Treatment of Hemorrhage from the Palm—Scrofulous Glands—Analysis of Milk—Another Drop in the Bucket—Absorption of Sequestra .................. 665–690

EDITORIAL:

Cholera Infantum .................. 691

Prize Essays and the Medical Association of Georgia .................. 694

BOOKS .................. 695

PAMPHLETS .................. 698

ITEMS .................. 699

